# Zika virus encephalitis in immunocompetent mice is dominated by innate immune cells and does not require T or B cells

**DOI:** 10.1186/s12974-019-1566-5

**Published:** 2019-09-11

**Authors:** Emina Hayashida, Zheng Lung Ling, Thomas M. Ashhurst, Barney Viengkhou, So Ri Jung, Pattama Songkhunawej, Phillip K. West, Nicholas J. C. King, Markus J. Hofer

**Affiliations:** 1 School of Life and Environmental Sciences, the Marie Bashir Institute for Infectious Diseases and Biosecurity, Charles Perkins Centre, and the Bosch Institute, The University of Sydney, Sydney, Australia; 2 Discipline of Pathology, the Marie Bashir Institute for Infectious Diseases and Biosecurity, the Bosch Institute, Charles Perkins Centre, School of Medical Sciences, Faculty of Medicine and Health, The University of Sydney, Sydney, Australia; 3 Sydney Cytometry Facility, The University of Sydney and the Centenary Institute, Sydney, Australia; 4 School of Life and Environmental Sciences, The University of Sydney, Molecular Bioscience Bldg., Maze Crescent G08, Sydney, NSW 2006 Australia

**Keywords:** Zika virus, Microglia, Monocyte, Neutrophil, Encephalitis, Innate immunity, Type I interferon, Adaptive immunity

## Abstract

**Background:**

Until the end of the twentieth century, Zika virus (ZIKV) was thought to cause a mostly mild, self-limiting disease in humans. However, as the geographic distribution of ZIKV has shifted, so too has its pathogenicity. Modern-day ZIKV infection is now known to cause encephalitis, acute disseminated encephalomyelitis, and Guillain-Barré syndrome in otherwise healthy adults. Nevertheless, the underlying pathogenetic mechanisms responsible for this shift in virulence remain unclear.

**Methods:**

Here, we investigated the contribution of the innate versus the adaptive immune response using a new mouse model involving intracranial infection of adult immunocompetent mice with a moderately low dose of ZIKV MR766. To determine the contribution of type I interferons (IFN-Is) and adaptive immune cells, we also studied mice deficient for the IFN-I receptor 1 (*Ifnar1*^*−/−*^) and recombination-activating gene 1 (*Rag1*^*−/−*^).

**Results:**

We show that intracranial infection with ZIKV resulted in lethal encephalitis. In wild-type mice, ZIKV remained restricted predominantly to the central nervous system (CNS) and infected neurons, whereas astrocytes and microglia were spared. Histological and molecular analysis revealed prominent activation of resident microglia and infiltrating monocytes that were accompanied by an expression of pro-inflammatory cytokines. The disease was independent of T and B cells. Importantly, unlike peripheral infection, IFN-Is modulated but did not protect from infection and lethal disease. Lack of IFN-I signaling resulted in spread of the virus, generalized inflammatory changes, and accelerated disease onset.

**Conclusions:**

Using intracranial infection of immunocompetent wild-type mice with ZIKV, we demonstrate that in contrast to the peripheral immune system, the CNS is susceptible to infection and responds to ZIKV by initiating an antiviral immune response. This response is dominated by resident microglia and infiltrating monocytes and macrophages but does not require T or B cells. Unlike in the periphery, IFN-Is in the CNS cannot prevent the establishment of infection. Our findings show that ZIKV encephalitis in mice is dependent on the innate immune response, and adaptive immune cells play at most a minor role in disease pathogenesis.

**Electronic supplementary material:**

The online version of this article (10.1186/s12974-019-1566-5) contains supplementary material, which is available to authorized users.

## Background

Zika virus (ZIKV), a member of the *Flaviviridae* family, was first isolated in 1947 in Uganda [1]. Although infections of humans had been reported since the 1950s in Africa, the disease was rare, mostly mild, and self-limiting [2, 3]. In 2007, an outbreak on Yap island in Micronesia resulted in approximately 70% of the population becoming infected by ZIKV [4]. This was followed by a second outbreak in 2013 in French Polynesia and rapid spread to most countries of South and Central America over a two year period (reviewed in [5, 6]). Critically, the spread of ZIKV was accompanied by a shift in the disease pattern and increased disease severity.

Modern-day ZIKV infection has been shown to cause microcephaly in fetuses [7–9] and neurological diseases in adults [10–16]. The pathological basis of fetal microcephaly has since been clarified. ZIKV is able to infect cells in the placenta and cross the placental barrier [17–20]. In the fetus, the virus shows a strong tropism for cells of the developing central nervous system (CNS), where it causes cell death of neural stem cells as well as precursors of astrocytes, oligodendrocytes, and microglia (reviewed in [21, 22]). By contrast, neurological diseases in otherwise healthy adults due to ZIKV infection are less well understood. ZIKV has been reported to cause encephalitis [15, 16], acute disseminated encephalomyelitis (ADEM) [14], and Guillain-Barré syndrome [10–13], an immune-mediated disorder of the peripheral nervous system in adults resulting in muscle weakness and, in rare cases, death (reviewed in [23]).

The susceptibility of humans to infection with ZIKV lies in the ability of the virus to potently suppress the antiviral immune response. Type I interferons (IFN-Is) are essential for activating the innate and adaptive immune responses by regulating the expression of several hundred interferon-stimulated genes (ISGs) (reviewed in [24, 25]). All IFN-Is bind to a heterodimeric cell surface receptor, termed IFNAR. Binding to the receptor activates a conserved Janus kinase (Jak)—signal transducers and activators of transcription (STAT) signaling pathway. Jak-mediated phosphorylation of STAT1 and STAT2 leads to the formation of a trimolecular complex with the interferon regulatory factor (IRF) 9 that binds to the promoter of ISGs resulting in their increased expression. The ZIKV nonstructural protein NS5 binds to human STAT2 and targets it for degradation, thus incapacitating an effective antiviral IFN-I response [26–28]. By contrast, murine STAT2 is not targeted by ZIKV and as a consequence, immunocompetent mice are largely resistant to infection [29–31]. Accordingly, most mouse studies have relied on either immune-compromised mice or extremely high virus doses to achieve productive infection. However, recently, a few studies using wild-type (WT) mice and moderate to low virus titers have been published. Pardy and colleagues have shown that peripheral infection of adult WT mice with 10^6^ PFU results in transient viremia and activation of innate and adaptive immune cells [32]. Similar findings have been obtained following intraperitoneal infection of 6-week-old WT mice; these showed activation of T cells but developed minor signs of disease only [33, 34]. Using a mouse-adapted, high-proliferating ZIKV strain, Gorman et al. were able to cause infection of 3-week-old immature transgenic mice that expressed human STAT2 instead of murine STAT2 (hSTAT2 KI mice) [35]. Unlike the studies in WT mice that had observed very mild signs of disease only, 30% of ZIKV-infected hSTAT2 KI mice died from an infection, further demonstrating the effectiveness of the IFN-I response in controlling the infection in mice. By contrast, to bypass the peripheral immune response, similar to George Dick’s results from 1952 [36], Nazerai et al. infected adult WT mice intracranially (i.c.) resulting in a lethal wasting disease [37]. However, neither study investigated the molecular or cellular basis of the disease, which remains unknown.

Here, we established and characterized a new fully immunocompetent mouse model to study ZIKV-induced CNS pathology. Our results show that following i.c. infection, ZIKV predominantly infects neurons in the adult CNS and induces lethal encephalitis that is dominated by activated resident microglia and infiltrating innate immune cells but is independent of adaptive immune cells. We further demonstrate that while IFN-Is were not able to prevent disease, they limited virus spread and modulated disease.

## Materials and methods

### Mice

*Ifnar1*^*−/−*^ and *Rag1*^*−/−*^ mice were previously described [38, 39]. Wild-type (WT) mice were purchased from Australian Bioresources (Moss Vale, NSW, AUS). All mice were on a C57BL/6 genetic background and bred under specific-pathogen-free conditions at the animal facility of the University of Sydney.

### Preparation of virus stock and intracranial infection of mice

ZIKV MR766 was obtained from the American Type Culture Collection (ATCC; ATCVR1838). The virus was propagated in Vero cells and infectivity determined by plaque-forming assay as previously described [40]. For infections, mice between 8 and 20 weeks of age were used. Mice were injected i.c. with 2 × 10^4^ PFU ZIKV in PBS through the postglenoid foramen as described in [41]. Sham-injected mice, which served as controls, received the same volume of PBS without virus. Prior to injection, mice were anesthetized with 100 μg ketamine and 1 μg xylazine per gram bodyweight. Following infection, mice were weighed and observed daily for the development of signs of disease. Clinical scores were determined by adding up individual scores based on an animal ethics-approved scoring scheme (rough fur = 1, prominent vertebral spinous processes, scapulae or pelvis = 2, hunched posture = 2, reduced activity = 2, tremor = 3, seizures = 4).

### Histology and immunohistochemistry

To determine pathological changes, mice were euthanized at the times shown, and the brains and peripheral organs (the liver and testes) were removed and fixed overnight in ice-cold 4% formaldehyde in PBS (pH 7.4). Following paraffin embedding, tissue sections (5 μm) were prepared and stained with hematoxylin and eosin (H&E). Immunohistochemistry was performed as described elsewhere [42]. The primary antibodies used were against GFAP (1:2000 dilution; Agilent, Mulgrave, VIC, AUS), Iba1 (1:1000 dilution; Wako, Osaka, Japan), and CD3 (1:100 dilution; Abcam, Melbourne, VIC, AUS). Sections were incubated successively with a biotinylated secondary antibody (1:200 dilution; Vector Laboratories, Burlingame, CA, USA) and streptavidin-coupled horseradish peroxidase (Vector Laboratories). Diaminobenzidine was applied as the peroxidase substrate, and sections were briefly counterstained with hematoxylin. For immunofluorescence, virus-infected cells were identified using a biotinylated primary antibody against flavivirus NS1 (20 μg/ml; clone 4G4) in combination with cell type-specific markers, GFAP (1:1000 dilution) for astrocytes, Iba1 (1:1000 dilution) for microglia and monocytes, or NeuN (1:200 dilution; Merck, Frenchs Forest, NSW, AUS) for neurons. Sections were subsequently incubated with a biotinylated secondary antibody (1:200 dilution, Invitrogen, North Ryde, NSW, AUS) and anti-rabbit-AF-488 (1:500 dilution, Invitrogen) and then SA-594 (1:500 dilution, Invitrogen). Stained sections were mounted with DAPI-containing media and examined under a DM4000B microscope (Leica, Wetzlar, Germany), and images were captured using a Spot Flex camera and Spot V4.5 software (Diagnostic Instruments).

### RNA isolation and RNase protection assay

Total RNA was isolated using TRIsure (Bioline, Alexandria, NSW, Australia). RPA against pro-inflammatory cytokine mRNA transcripts, IFN-α1, IFN-β, TNF, IL-1α, IL-1β, IL-2, IL-3, IL-4, IL-5, IL-6, and IFN-γ, was performed as previously described [42]. The probe for ZIKV was synthesized by reverse transcription-PCR using forward primer 5′-AATGAATTCAGCTAACAACAG TATCAACAGGT-3′ and reverse primer 5′-AATAAGCTTAAGGGGTTT ACA CGGGCTAC-3′ that target the 5′UTR and capsid gene of ZIKV (reference sequence KU963574.2), cloned, and verified by sequencing analysis as previously described [43]. Following RPA, RNA levels were quantified from autoradiographs by densitometry using NIH Image software (version 1.47) as described previously [43, 44].

### In situ hybridization and dual-label in situ hybridization/histochemistry

ISH for ZIKV RNA with a probe synthesized using forward primer 5′-AATGAATTCACCCAAAGAAGAAATCCGGAG-3′ and reverse primer 5′-AATAAGCTTCCAGTGATGGCTTGATTGCT-3′ was performed as described previously [45]. Briefly, 5-μm-thick paraffin-embedded sections were pre-treated and incubated with a ^33^P-labeled ZIKV cRNA probe overnight. Sections were then washed, dehydrated, and exposed to Kodak Biomax MR film (Sigma-Aldrich, Castle Hill, NSW, AUS). For dual-label ISH/HC, following incubation with a ^33^P-labeled ZIKV cRNA probe, washing, and dehydration, sections were reacted with antibodies to detect neurons (NeuN, 1:200 dilution) or astrocytes (GFAP, 1:1000 dilution). Microglia, infiltrating monocytes/macrophages and blood vessels, were detected with biotinylated lectin from *Lycopersicon esculentum* (1:50 dilution, Sigma-Aldrich). Bound antibody or lectin was detected using Vectastain ABC kits (Vector Laboratories) and diaminobenzidine-H_2_O_2_ reagent (Vector Laboratories) as the immunoperoxidase substrate.

### Cell isolation and flow cytometry

Leukocytes were isolated from the CNS as previously described [46]. Harvested cells were blocked for non-specific binding with F_c_ block (BioLegend, San Diego, CA, USA) in UV-excitable fixable live/dead blue stain (1:500 dilution; Thermo Fisher Scientific). Cells were then incubated with fluorochrome-conjugated antibodies to specifically stain surface markers. Antibodies used were αCD11b-BUV395 (clone: M1/70; BD Biosciences), αB220-BUV737 (clone: RA3-6B2; BD Biosciences), αLy6C-BV605 (clone: HK1.4; Biolegend), αLy6G-BV650 (clone: 1A8; Biolegend), αCD8α-BV711 (clone: 53-6.7; Biolegend), αCD11c-BV785 (clone: N418; Biolegend), αF4/80-PE (clone: BM8; Biolegend), αCD3ε-PE/CF594 (clone: 145-2C11; BD Biosciences), αNK1.1-PE/Cy7 (clone: PK136; Biolegend), αCD45-AF700 (clone: 30-F11; Biolegend), αCD4-APC (clone: GK1.5; Biolegend), and αI-A/I-E-APC/Cy7 (clone: M5/114.15.2; Biolegend). Cell types were identified based on the expression of CD45^lo^, CD11b^+^, and Ly6c^lo^ for microglia; CD45^hi^, CD11b^+^, and Ly6c^hi^ for inflammatory macrophages; CD11b^hi^ and Ly6G^+^ for neutrophils; CD45^+^ NK1.1^+^ and CD3ε^−^ for NK cells; CD3ε^+^ and CD4^+^ for CD4^+^ T cells; CD3ε^+^ and CD8^+^ for CD8^+^ T cells; and B220^+^, NK1.1^−^, and CD11c^−^ for B cells (Additional file 1). Data were acquired on the Becton Dickinson LSR-II 10-Laser flow cytometer (BD Biosciences). Compensation was performed using eBioscience™ UltraComp eBeads (Thermo Fisher Scientific) conjugated with fluorescent antibodies for each channel. Data analysis was performed using FlowJo × 10.0.7 software (Tree Star, Ashland, OR, USA).

### Computational analysis of cytometry data

Live CD45^+^ cells were manually gated and exported as CSV-channel files, which transforms the data in such a way that allows logarithmic data to be plotted on a linear scale. Computational analysis of data was performed using the CAPX analysis pipeline, with instructions provided at [47] and source code provided at (www.github.com/sydneycytometry/tSNEplots). CSV-channel files were imported into R using CAPX, where keywords indicating the sample and group names were embedded, before samples were selectively downsampled and merged. The FlowSOM algorithm [48] was then run on the merged dataset to perform clustering, where every cell was assigned to a specific cluster. Subsequently, the data was randomly downsampled (to a maximum of 8 × 10^4^ cells, by downsampling each individual sample or group by the same proportion) in preparation for visualization using t-Distributed Stochastic Neighbor Embedding (tSNE) [49–51]. A series of tSNE plots colored by the level of expression of each marker for every sample were generated automatically by the CAPX pipeline (separate script available at (www.github.com/sydneycytometry/tSNEplots)), and the resulting tSNE plots were used to explore and identify populations in the dataset. All populations were identified through an exploration of the colored tSNE plots, overlay of manual gates, and comparison of FlowSOM clusters. Custom R scripts were then used to add population names to the dataset and provide summarized data. Because tSNE was run on a subset of the large clustered dataset, clusters identified using tSNE analysis could be isolated from the original large dataset for numerical and statistical analysis. Numerical comparisons and plotting were performed using the AutoGraph script found at (https://github.com/sydneycytometry/AutoGraph) or Prism software (v7). Statistical comparisons of two groups were performed using a Mann-Whitney-Wilcoxon test for non-Gaussian data (also referred to as a “Wilcox test” in R) using Prism (v7). The overall variance of the dataset was assessed using a Kruskal-Wallis test for non-Gaussian data.

Heatmaps showing the fold-change of the number of cells per cluster in each sample, relative to the average of WT mock-infected samples, were generated using a custom R script. Fold-change was plotted in log_2_ and colored black (0 in log_2_, no change), red/yellow (an increase in fold change in log_2_, > 0 to greater than or equal to the maximum value indicated on the scale bar), or blue (a decrease in fold change in log_2_, < 0 to less than or equal to the minimum value indicated on the scale bar). Columns and rows were either ordered manually or clustered together based on similarity, indicated by the colored bars that group columns, determined by Euclidean distance.

### Statistics

Details of statistical analysis are given in the respective section of the materials and methods or the figure legends. The software Prism version 7 for Mac OS X (GraphPad Software, La Jolla, CA, USA) was used for all statistical analyses.

## Results

### Intracranial infection of wild-type, *Rag1*^*−/−*^, and *Ifnar1*^*−/−*^ mice with ZIKV is lethal

**Fig. 1:**
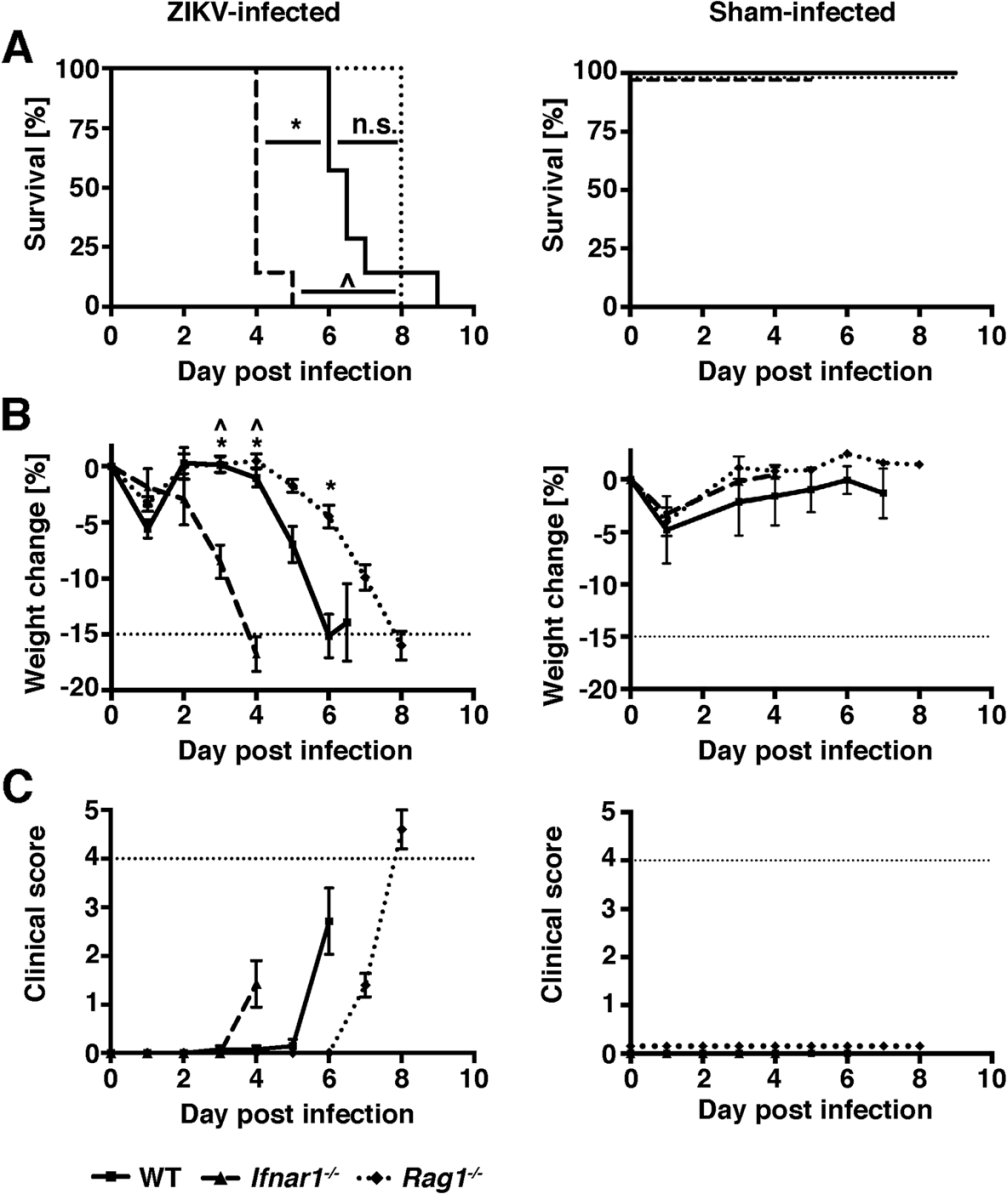
ZIKV causes a lethal disease in WT, *Rag1*^*−/−*^, and *Ifnar1*^*−/−*^ mice following intracranial infection. WT (*n* = 13, solid lines), *Ifnar1*^*−/−*^ (*n* = 7, dashed lines), and *Rag1*^*−/−*^ (*n* = 8, dotted lines) mice were infected i.c. with ZIKV MR766. Sham-injected mice (*n* = 3 per genotype) received virus diluent only. Survival (**a**), weight loss (**b**), and clinical scores (**c**) were determined daily. Mean ± SEM are shown, and additional horizontal dotted lines indicate humane endpoint criteria as approved by the University of Sydney Animal Ethics Committee. For **a**, *p < 0.05 compared with WT, ^*p* < 0.05 compared with *Ifnar1*^*−/−*^, and n.s. = not significant as determined by Log-rank test. For **b**, *p < 0.05 when compared to the corresponding time point in WT mice and ^*p* < 0.05 when compared to the corresponding time point in *Rag1*^*−/−*^ mice, as determined by two-way ANOVA. One of two representative experiments is shown

To determine if ZIKV causes disease in immunocompetent mice after bypassing the peripheral immune system, WT mice were infected i.c. with a moderately low dose of 2 × 10^4^ PFU. We used the mouse-adapted strain of the African lineage, ZIKV MR766, since murine cells are permissible to this strain and low to moderate doses are sufficient to elicit immune responses in vivo and in vitro*.* Following i.c. infection, all WT mice had to be euthanized between day 6 and 9 post infection, due to a weight loss of 15% and/or a clinical score of 4 or more, whereas none of the sham-injected mice showed a significant loss of weight or developed signs of disease (Fig. 1a–c). Signs of disease exhibited in ZIKV-infected mice included a hunched posture and reduced activity. However, seizures were not observed. This experiment demonstrated that once ZIKV gains entry to the CNS, it is able to induce lethal disease in the presence of a normal immune system.

To elucidate the disease mechanism and to clarify if key components of the antiviral host response contributed to the disease, we infected *Rag1*^*−/−*^ mice, which are deficient in mature T and B cells, and *Ifnar1*^*−/−*^ mice, which are unresponsive to all IFN-Is. As expected, sham-injected mice showed mild weight loss and no signs of disease (Fig. 1a–c). When infected peripherally, T and B cells do not play a significant role in host resistance to ZIKV, and *Rag1*^−/−^ mice do not develop significant weight loss or disease [52]. By contrast, i.c. infection of *Rag1*^*−/−*^ mice caused a lethal disease that was similar to the disease seen in WT mice (Fig. 1a–c). Weight loss and onset of signs of disease was slightly delayed in *Rag1*^*−/−*^ mice compared with WT mice, but all *Rag1*^*−/−*^ mice had to be euthanized on day 8 post infection. In contrast to adaptive immunity, IFN-Is are critical to prevent ZIKV disease in mice [29, 31, 33, 53, 54]. In line with this, i.c. infection of *Ifnar1*^*−/−*^ mice resulted in a rapid onset of weight loss from day 2 post infection. Critical weight loss necessitated euthanasia of all *Ifnar1*^*−/−*^ mice between days 4 and 5 post infection. These mice had to be euthanized only due to weight loss, which made the survival of *Ifnar1*^*−/−*^ mice significantly shorter than in WT or *Rag1*^*−/−*^ mice. Other signs of disease in *Ifnar1*^*−/−*^ mice were mild and limited to hunched posture and reduced activity.

### In wild-type and *Rag1*^*−/−*^ mice, ZIKV is limited to the CNS and predominantly infects neurons but spreads in *Ifnar1*^*−/−*^ mice

**Fig. 2:**
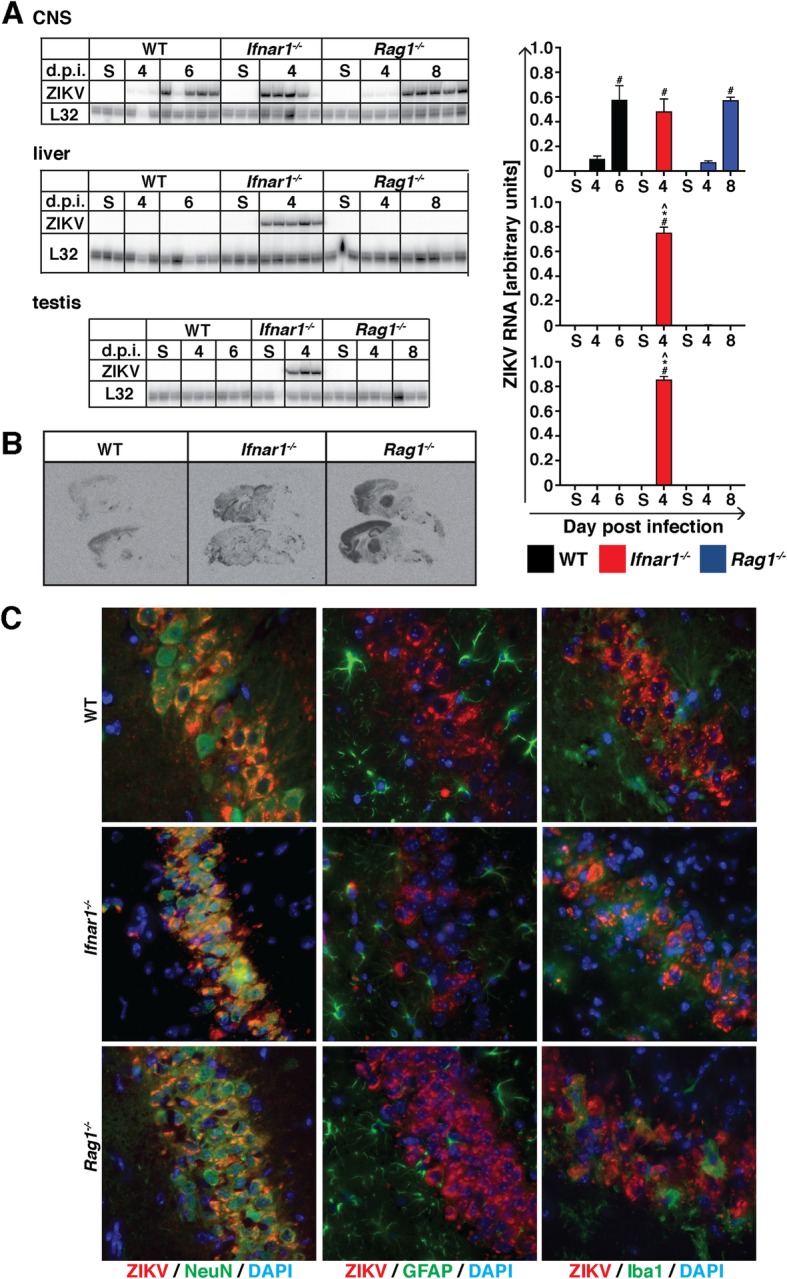
In WT and *Rag1*^*−/−*^ mice but not *Ifnar1*^*−/−*^ mice, ZIKV is restricted to the CNS following intracranial infection. **a** Following i.c. infection, ZIKV RNA is detectable in the CNS of WT (black), *Ifnar1*^*−/−*^ (red) and *Rag1*^*−/−*^ mice (blue). However, ZIKV RNA is only detectable in the livers and testes of *Ifnar1*^*−/−*^ mice but not WT or *Rag1*^*−/−*^ mice. *p < 0.05 when compared with the corresponding time point in WT mice, ^*p* < 0.05 when compared with the corresponding time point in *Ifnar1*^*−/−*^ and *Rag1*^*−/−*^ mice, and #*p* < 0.05 compared to sham-injected mice as determined by one-way ANOVA with Tukey’s multiple comparisons test. **b** In situ hybridization shows that at peak disease, ZIKV predominantly infects the cortex, hippocampus, and thalamus of i.c.-infected WT mice. At peak disease, in *Ifnar1*^*−/−*^ and *Rag1*^*−/−*^ mice, the virus spread to the brain stem and in *Ifnar1*^*−/−*^ mice, ZIKV also infects the cerebellum. **c** Dual-label immunofluorescence (DL-IF) was performed with antibodies against flavivirus NS1 and markers to detect neurons (NeuN), astrocytes (GFAP), or microglia (Iba1). DAPI was used to stain nuclei. Co-localization of ZIKV NS1 protein and cell type marker is evident by orange/yellow color. DL-IF shows that ZIKV infects predominantly neurons but not astrocytes or microglia independent of the genotype. Representative images of a WT, *Ifnar1*^*−/−*^, and *Rag1*^*−/−*^ hippocampus are shown

Next, we determined the spread of ZIKV by RNase protection assay (RPA). In addition to the CNS, we also assessed viral RNA levels in the liver and testis, as both organs had previously been shown to be a target of ZIKV [29, 33, 55]. In WT mice, ZIKV RNA was detectable in the CNS at low levels at day 4 and had increased by day 6 post infection (Fig. 2a). In line with published observations that ZIKV is rapidly eliminated following peripheral infection, no viral RNA was detectable in the liver or testis of infected WT mice at either day 4 or 6 post infection. By contrast, in *Ifnar1*^*−/−*^ mice, ZIKV RNA was detectable at high levels in the CNS, liver, and testis at day 4 post infection (Fig. 2a). In *Rag1*^*−/−*^ mice, ZIKV RNA was detectable only in the CNS but not in peripheral organs, and relative levels in the CNS were similar to those seen in WT mice. This demonstrates that IFN-Is but not T or B cells are required to restrict ZIKV spread and replication.

We investigated the spatial distribution of ZIKV RNA in the CNS of mice by in situ hybridization (ISH) at peak disease, i.e., day 6 for WT, day 4 for *Ifnar1*^*−/−*^, and day 8 post infection for *Rag1*^*−/−*^ mice. In all three genotypes, ZIKV RNA was present in the cortex, hippocampus, and thalamic region and to a lesser extent in the midbrain and brainstem (Fig. 2b). In addition, in *Ifnar1*^*−/−*^ and *Rag1*^*−/−*^ mice, ZIKV RNA was also observed in other areas of the cerebrum, and in the midbrain and brain stem, and in *Ifnar1*^*−/−*^ mice also in the cerebellum. Overall, while virus distribution was more extensive in *Rag1*^*−/−*^ mice than WT mice, ZIKV-infected brain regions were well defined in both genotypes, whereas in *Ifnar1*^*−/−*^ mice virus spread appeared more diffuse. Thus, in the absence of a functional IFN-I system, there is an increased virus spread within the CNS, as well as virus spread to other organs.

To identify the cellular target of ZIKV in the CNS, we performed immunofluorescence with an antibody targeting flavivirus NS1. Independent of the genotype of mice, ZIKV infected neurons primarily (Fig. 2c). By contrast, astrocytes and microglia were only rarely NS1-positive in WT, *Rag1*^*−/−*^, and *Ifnar1*^*−/−*^ mice. This was also seen by dual-label ISH, where ZIKV RNA co-localized to NeuN-positive neurons but rarely to GFAP-positive astrocytes or tomato lectin-positive microglia (Additional file 2).

### ZIKV infection causes prominent perivascular encephalitis in wild-type and *Rag1*^*−/−*^ mice and diffuse infiltration by polymorphonucleated cells in *Ifnar1*^*−/−*^ mice

**Fig. 3:**
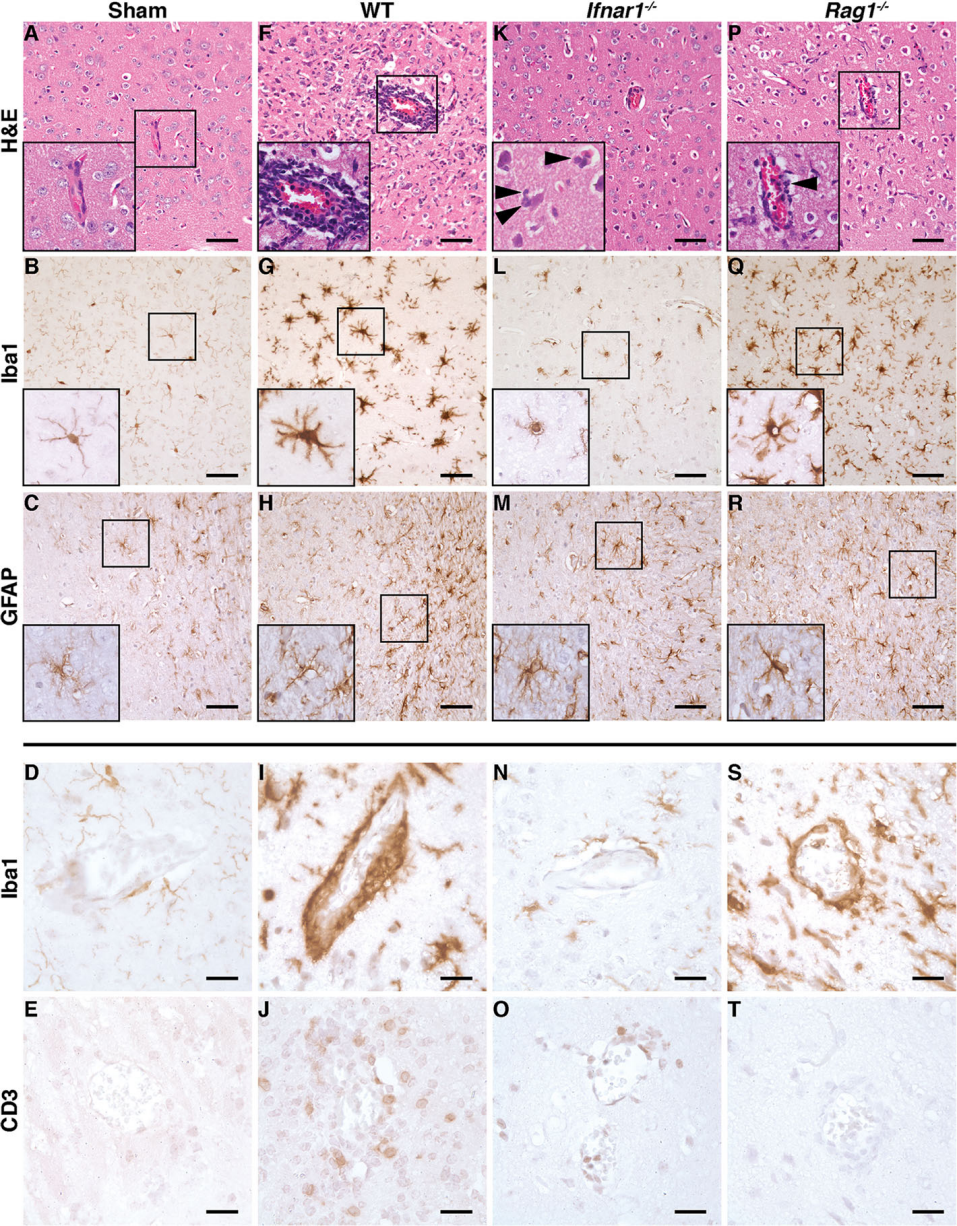
Following i.c. infection, WT and *Rag1*^*−/−*^ mice but not *Ifnar1*^*−/−*^ mice develop encephalitis. **a**, **f**, **k**, **p** H&E-stained section from the CNS of sham-injected WT mice and mice infected with ZIKV at peak disease. **a** No inflammatory infiltrates were seen in sham-injected mice. The CNS of ZIKV-infected WT mice at day 6 post infection showed pronounced encephalitis with perivascular cuffing (**f**) that was less extensive in *Rag1*^−/−^ mice at day 8 post infection (**p**). By contrast, *Ifnar1*^*−/−*^ mice (**k**) showed only few infiltrating leukocytes, predominantly PMNs (inset, arrowheads) at peak disease (day 4 post infection). **b**, **d**, **g, i**, **l**, **n**, **q**, **s** Immunohistochemistry for the microglia marker Iba1. Compared with sham-injected WT mice (**b**), the CNS of diseased WT (**g**) and *Rag1*^*−/−*^ mice (**q**) revealed an increased staining intensity for Iba1 and microglia had short and thickened process characteristics for activation. Further, monocytes/macrophages formed perivascular cuffs in diseased WT (**i**) and *Rag1*^*−/−*^ mice (**s**). The brains from infected *Ifnar1*^*−/−*^ mice (**l**, **n**) showed no gross differences compared to sham controls (**b**, **d**). **c**, **h**, **m**, **r** Immunohistochemistry for the astrocyte marker GFAP. Compared with sham-injected WT mice (**c**), moderate astrogliosis was observed in the CNS of all infected mice independent of the genotype. **e**, **j**, **o**, **t** Immunohistochemistry for the T cell marker CD3. No T cells were seen in sham-injected mice (**e**), whereas diseased WT mice showed large numbers of CD3^+^ T cells in the perivascular space and diffusely infiltrating the parenchyma (**j**). Some CD3^+^ T cells were seen in diseased *Ifnar1*^*−/−*^ mice (**o**). Expectedly, a stain for CD3 was negative in diseased *Rag1*^*−/−*^ mice (**t**). **a**, **b**, **c**, **f**, **g**, **h**, **k**, **l**, **m**, **p**, **q**, **r**: scale bar = 50 μm; **d**, **e**, **i**, **j**, **n**, **o**, **s**, **t**: scale bar = 20 μm

To assess whether i.c. infection with ZIKV results in tissue pathology, we analyzed tissue sections from the CNS of infected mice at peak disease by routine histology and immunohistochemistry. Compared with sham-injected controls (Fig. 3a–e), ZIKV infection at peak disease (day 6) was associated with extensive infiltration of leukocytes that had accumulated around blood vessels in the CNS parenchyma of WT mice (Fig. 3f). This was accompanied by increased staining of microglia with Iba1 (Fig. 3g) compared with sham-injected controls (Fig. 3b). Also, microglia from infected WT mice had thick and short cellular processes characteristic of activated microglia (i.e., microgliosis). Astrocyte activation (i.e., astrogliosis) was only moderate in the CNS of infected WT mice (Fig. 3h). Further, Iba1 and CD3 stains revealed numerous monocytes/macrophages (Fig. 3i) and T cells (Fig. 3j), respectively, surrounding the intracerebral blood vessels. In contrast to infected and diseased WT mice, the CNS of infected *Ifnar1*^*−/−*^ mice at peak disease (day 4) showed only minor inflammatory infiltrates (Fig. 3k). However, unlike WT mice, the brain parenchyma of infected *Ifnar1*^*−/−*^ mice contained large numbers of diffusely distributed polymorphonucleated cells (PMNs) (Fig. 3k, arrowheads in the inset). Also, microgliosis (Fig. 3l) and astrogliosis (Fig. 3m) was almost completely absent in the CNS of infected *Ifnar1*^*−/−*^ mice. In line with this, only few monocytes/macrophages (Fig. 3n) and some T cells (Fig. 3o) were seen surrounding blood vessels. The brains from infected *Rag1*^*−/−*^ mice at peak disease (day 8) showed a phenotype intermediate to that observed in diseased WT and *Ifnar1*^*−/−*^ mice (Fig. 3p–t) with moderate inflammatory changes and few PMNs (Fig. 3p, arrowhead in the inset). Expectedly, T cells were absent from the brain of infected *Rag1*^*−/−*^ mice (Fig. 3t).

The liver and testis were also examined for histopathological changes at peak disease in all genotypes. No observable features of pathology were present in the liver or the testis of WT mice following i.c. infection with ZIKV (not shown). In two out of seven *Ifnar1*^*−/−*^ mice, infiltrating leukocytes were present around the blood vessels of the portal triad. Infection of the liver was characterized by hepatocytes with dark or enlarged nuclei, commonly observed in inflammatory responses (not shown). Furthermore, in *Ifnar1*^*−/−*^ mice, the epithelial layer of the testis was disordered and had a noticeable lack of spermatids compared to sham-injected controls (not shown). PMNs were observed in some mice. The liver or testis of *Rag1*^*−/−*^ mice showed no overt morphological changes, except for occasional cells that resembled PMNs.

### Expression of pro-inflammatory cytokine genes correlates with virus spread in wild-type and *Rag1*^*−/−*^ mice but not in *Ifnar1*^*−/−*^ mice

**Fig. 4:**
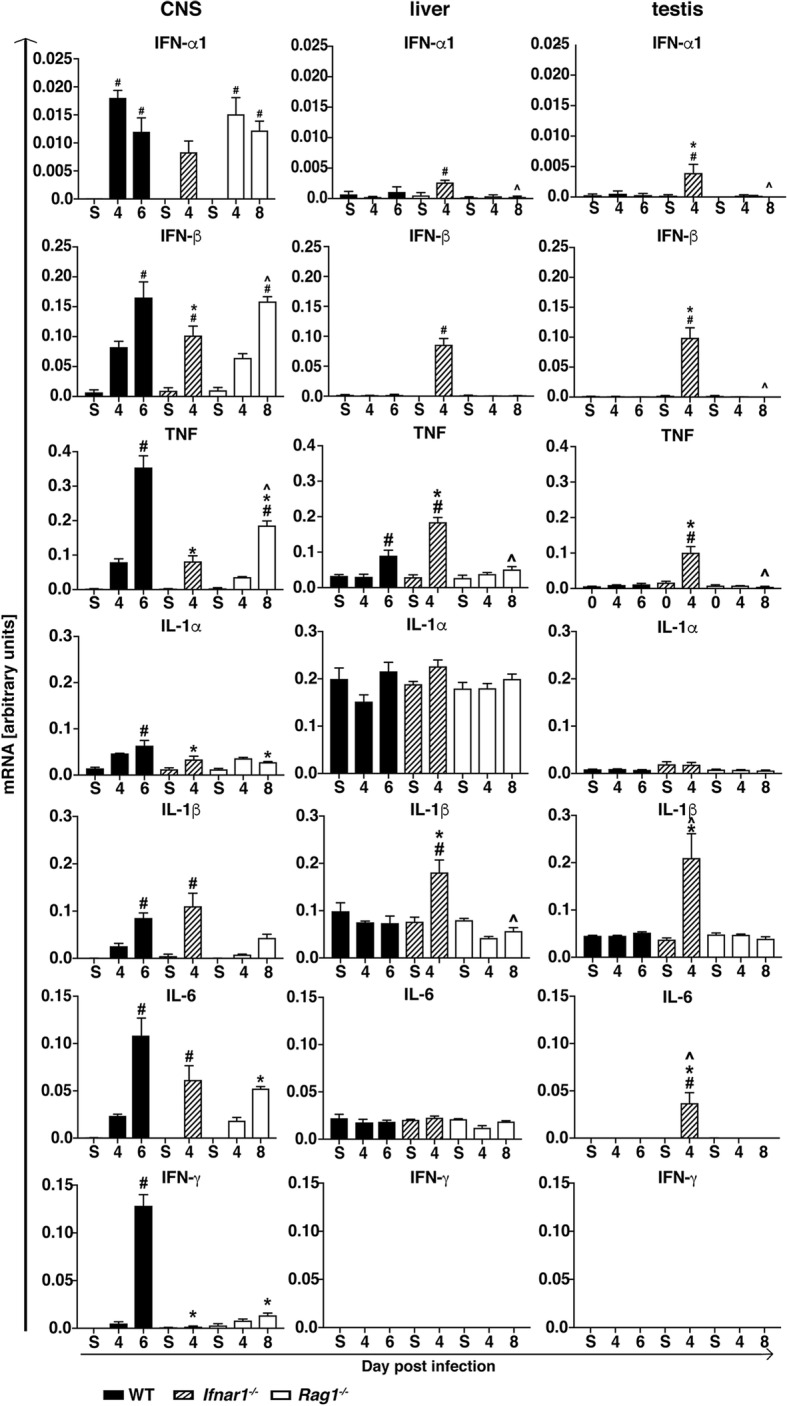
Expression of pro-inflammatory cytokine genes in the CNS, liver, and testis of WT, *Ifnar1*^*−/−*^, and *Rag1*^*−/−*^ mice. Densitometric quantifications of RPAs for IFN-α1, IFN-β, TNF, IL-1α, IL-1β, IL-6, and IFN-γ mRNA. Cytokine mRNA levels were normalized to the housekeeping gene *L32*. Values are shown as mean ± SEM. #*p* < 0.05 when compared with sham-injected mice, *p < 0.05 when compared with day 6 (peak disease) in WT mice, ^*p* < 0.05 when compared with day 4 (peak disease) in *Ifnar1*^−/−^ mice, as determined by one-way ANOVA

We determined the expression of pro-inflammatory cytokine genes in the CNS, liver, and testis of mice. In the CNS of WT mice, increased IFN-α1, IFN-β, TNF, IL-1α, IL-1β, IL-6, and IFN-γ mRNA levels were seen at day 4 post infection, and with the exception of IFN-α1, which decreased slightly, cytokine mRNA levels increased further by day 6 post infection (Fig. 4). In the livers of infected WT mice, only TNF mRNA was significantly increased at day 6 post infection, whereas mRNA levels for the other cytokine genes were comparable to the sham-injected mice and IFN-γ mRNA was undetectable. In the testis, no differences were seen between sham-injected and ZIKV-infected mice. Finally, mRNA for IL-2, IL-3, IL-4, and IL-5 was not detectable in any organs or time points in WT mice (data not shown).

In the CNS of *Ifnar1*^*−/−*^ mice, IFN-α1, IFN-β, TNF, IL-1α, IL-1β, and IL-6 mRNA levels were increased at day 4 post infection when compared with sham-injected controls (Fig. 4). IFN-γ mRNA was only marginally detectable. In addition, in the livers, IFN-α1, IFN-β, TNF, and IL-1β mRNA and, in the testes, IFN-α1, IFN-β, TNF, IL-1β, and IL-6 mRNA levels increased following infection of *Ifnar1*^*−/−*^ mice. Notably, at peak disease, IFN-β, TNF, and IL-1α mRNA levels were significantly lower in the CNS of *Ifnar1*^*−/−*^ mice than in WT mice, whereas in the liver, TNF and IL-1β mRNA and, in the testis, TNF, IL-1β, and IL-6 mRNA were significantly higher, concordant with the presence of virus RNA (Fig. 2a). Also, IFN-γ mRNA was not detectable in the livers or testes of sham-injected or ZIKV-infected *Ifnar1*^*−/−*^ mice. Comparable to WT mice, IL-2, IL-3, IL-4, and IL-5 mRNA was undetectable in the CNS, liver, or testis of *Ifnar1*^*−/−*^ mice (data not shown).

Similar to WT mice, in the CNS of *Rag1*^*−/−*^ mice IFN-α1, IFN-β, TNF, IL-1α, IL-1β, IL-6, and IFN-γ mRNA levels increased following i.c. infection with ZIKV (Fig. 4), and IL-2, IL-3, IL-4, and IL-5 mRNA was not detectable (data not shown). Interestingly, TNF, IL-1α, IL-6, and IFN-γ mRNA levels were significantly lower in the CNS of *Rag1*^*−/−*^ mice compared with WT mice at peak disease, correlating with the milder histological changes in these mice, while IFN-α1 and IFN-β mRNA levels were comparable. None of the cytokine genes investigated showed significantly increased mRNA levels in the liver and testis following infection.

### Inflammatory infiltrates in the CNS are dominated by inflammatory macrophages, NK cells, and resident microglia in wild-type mice and by neutrophils in *Ifnar1*^*−/−*^ mice

**Fig. 5:**
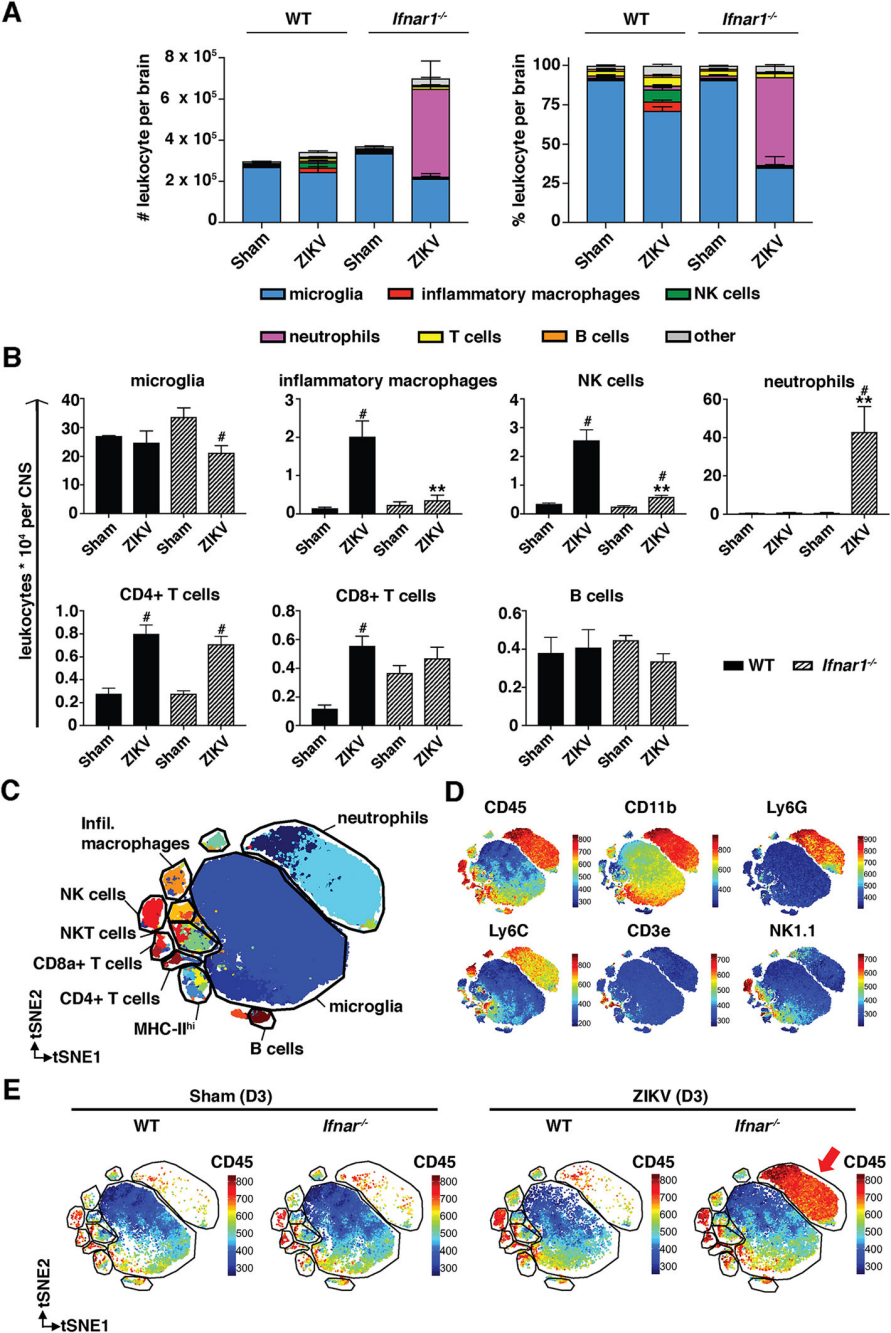
Identification and quantification of CNS-resident microglia and infiltrating leukocytes in infected WT and *Ifnar1*^*−/−*^ mice. **a** Number and percentage of microglia and infiltrating leukocytes in the CNS of sham- and ZIKV-injected WT and *Ifnar1*^*−/−*^ mice as determined by flow cytometry (*n* = 3 for sham and *n* = 5 for ZIKV-infected). **b** Numbers of microglia and leukocyte subpopulations in the CNS of sham- and ZIKV-injected WT and *Ifnar1*^*−/−*^ mice. Microglia, inflammatory macrophages, NK cells, neutrophils, CD4+ T cells, CD8+ T cells, and B cells were identified based on the presence of surface markers. **c** tSNE plot of entire dataset (WT and *Ifnar1*^*−/−*^, infected and sham-injected) colored by FlowSOM cluster identities, where every point is a single cell. **d** tSNE plots of the same dataset colored by the expression of various markers. **e** tSNE plots of the same dataset, split into sham-infected WT and *Ifna1r*^*−/−*^ mice, and ZIKV-infected WT and *Ifnar1*^*−/−*^ mice; colored by the expression of CD45. **p < 0.01 when compared with infected WT mice, #*p* < 0.05 compared with respective sham-injected mice as determined by Mann-Whitney test for panel **b**

Histological analysis had shown prominent perivascular cuffs in the CNS of WT mice and mostly diffuse infiltrating PMNs in *Ifnar1*^*−/−*^ mice, following infection with ZIKV (Fig. 3d, g). To further characterize the infiltrating leukocytes, we isolated leukocytes from the CNS of sham-injected and ZIKV-infected mice at day 3 post infection. This early time point was necessary as *Ifnar1*^*−/−*^ mice became sick at that day and had to be euthanized. Therefore, all mice were euthanized on day 3 post infection for accurate comparison. Following i.c. infection of WT mice, there was only a small increase in the total number of leukocytes (including microglia) in the CNS (Fig. 5a). However, the proportion of inflammatory macrophages and natural killer (NK) cells was significantly increased in the CNS of infected WT mice, compared with sham-injected mice (Fig. 5a, b). In agreement with the immunohistochemistry (Fig. 3j), a significant increase in the numbers of CD4^+^ T cells and CD8^+^ T cells was seen, while the number of microglia and B cells did not significantly change (Fig. 5b). Only very few neutrophils were detected in the CNS of sham-injected or ZIKV-infected WT mice.

In contrast to WT mice, in *Ifnar1*^*−/−*^ mice, the total number of leukocytes had almost doubled by day 3 post infection (Fig. 5a). Both by proportion and absolute numbers, neutrophils were by far the most abundant leukocyte cell type in the CNS of ZIKV-infected *Ifnar1*^*−/−*^ mice (Fig. 5a, b). Further, neutrophils and CD4^+^ T cells were the only cell type that increased significantly in the brains of *Ifnar1*^*−/−*^ mice following infection. When comparing infected WT and *Ifnar1*^*−/−*^ mice, the number of inflammatory macrophages was significantly higher in the CNS of WT mice, whereas the number of neutrophils was significantly higher in *Ifnar1*^*−/−*^ mice. Of note, the increase in CD4^+^ T cells and the low number of inflammatory macrophages identified by flow cytometry fits well to the immunohistochemical findings that showed an increased number of CD3^+^ T cells (Fig. 3o) but only few monocytes/macrophages surrounding blood vessels (Fig. 3n).

To confirm our results, we performed computational analysis of the dataset through clustering (using FlowSOM) and dimensionality reduction (using tSNE), using the CAPX script in R [47]. Using this approach, we were able to identify the same subsets of cells as in the manual gating analysis (Fig. 5c, d, and Additional file 3), including microglia, infiltrating macrophages, neutrophils, CD4^+^ T cells, CD8^+^ T cells, NK cells, NKT cells, and B cells, in addition to other phenotypes. When applying the gating strategy on the clustered cells, we found overall agreement between the cell identities determined by manual gating and those determined by exploration of the clustering results (Additional file 1: Figure S1B). The overall changes in these populations were consistent with those identified by manual gating (Additional file 4a, b) with the most striking change being the appearance of a large number of neutrophils in the ZIKV-infected *Ifnar1*^*−/−*^ brains (Fig. 5e). When investigating these cells more closely, we found that a large number of these cells exhibited a less mature phenotype of neutrophils, with lower Ly6G and CD11b expression (Additional file 3 A and B, and C, cluster #12).

## Discussion

As the geographic distribution of ZIKV has shifted, so too has its pathogenicity. In contrast to the mostly mild, self-limiting disease reported in the 1950s, modern-day ZIKV infection is known to cause severe and lethal diseases of the CNS and peripheral nervous system in fetuses as well as in immunocompetent adults [7–16]. However, the underlying pathogenetic mechanisms responsible for the increased virulence remain unclear. Here, we report a new mouse model using i.c. infection of adult immunocompetent mice with a moderately low virus dose. Unlike most previously reported models, the combination of an immunocompetent host and comparatively low infectious dose provides a good model to study the immunopathology of ZIKV infections in the CNS.

The efficiency of the peripheral immune system and in particular the IFN-Is in mice effectively prevents infection of the CNS following peripheral infection with ZIKV [31]. To bypass this, we chose to infect animals intracranially. Unlike peripheral infection of immunocompetent WT mice, which results in rapid virus elimination in the absence of severe disease [29–31, 33], i.c. infection was lethal in 100% of the mice, demonstrating that the IFN-I response in the CNS is insufficient to prevent the establishment of a productive infection. Recently, Nazerai et al., using the same virus strain and a similar dose to us, also reported that i.c. infection of immunocompetent mice results in lethal disease [37]. Onset of weight loss at day 4 post infection and death by day 8 post infection were comparable to our results. Interestingly, compared with C57Bl/6 mice, Balb/c mice were partially protected from death following infection with a very low virus dose of 10^2^ PFU [37] suggesting that differences between mouse strains may affect outcome of infection. However, Nazerai et al. did not investigate any underlying cellular or molecular mechanisms. By contrast, in our study, the pathological hallmark of the lethal disease was a pronounced encephalitis and microgliosis that was most prominent in the cortex, hippocampus, and thalamic regions. This coincided with the presence of ZIKV being largely limited to these regions. Interestingly, we observed almost exclusive infection of neurons, whereas other cell types of the CNS were mostly spared. This neuronal tropism was similar to findings by others, who found neurons to be primarily targeted when infecting immature mice i.c. [56]. By contrast, peripheral infection of newborn WT mice results initially in the infection of astrocytes, followed by neurons [57], and this difference may be due to the route of infection. Following peripheral infection, ZIKV gains access to the CNS through the immature blood-brain barrier, which is lined with astrocyte processes. Despite the slight difference in the cellular distribution between our study and the experiments done by van den Pol et al. [57], both studies observed neuronal infection primarily in the hippocampus, cortex, and thalamus, whereas Manangeeswaran et al. found that ZIKV primarily targeted the cerebellum and hippocampus following subcutaneous infection of 1-day-old mice [58]. The reason for this difference is unknown but may be due to the age of animals or the virus strain used, although van den Pol et al. [57] and Manangeeswaran et al. [58] both used Asian lineage strains. Our results add to these findings that the cellular tropism of ZIKV in the CNS is independent of the lineage. Of note, the clinical course, neurotropism, and virus spread in our study were similar to those reported for CNS infection models with other neurotropic members of the *Flaviviridae* family, including West Nile virus (WNV) [46, 59] and dengue virus (DENV) [60]. It is tempting to speculate that this is due to the same cell entry receptors being implicated for all three viruses to contribute to CNS infection [61, 62], suggesting a conserved pathogenesis amongst the neurotropic *Flaviviridae*.

As mentioned above, the pathological hallmark of the disease in i.c.-infected WT mice was the pronounced microgliosis and infiltration of the CNS by inflammatory macrophages and NK cells. Our findings of a nearly identical clinical course, comparable virus RNA levels, virus restriction to the CNS, and histological pattern in *Rag1*^*−/−*^ mice that lack mature T and B cells, suggest a minor role for adaptive immunity in ZIKV pathogenesis. In line with this, depletion of CD4^+^ or CD8^+^ cells had no significant effect in WT mice infected peripherally where ZIKV is effectively controlled [52]. It is worth noting that despite being dispensable, CD4^+^ and CD8^+^ T cells proliferate and become activated following infection of mice with ZIKV [32, 34, 52], and both T and B cells are critical to protect ZIKV-immune mice from intracranial challenge with the virus [37]. Further, in line with a minor role for T and B cells following intracranial ZIKV infection, we observed only slight differences in the clinical course and somewhat more widespread infection in the CNS—in the absence of overall increased virus RNA levels—in *Rag1*^*−/−*^ mice compared with WT mice. Although the exact role of adaptive immunity in the antiviral host response against ZIKV will need to be clarified further, a recent study has shown that IFN-γ, which is primarily produced by T cells and NKT cells, is critical in limiting virus replication in the CNS following peripheral infection [63]. In line with this, IFN-γ mRNA levels were significantly reduced at peak disease in *Rag1*^*−/−*^ mice compared with WT mice, coinciding with virus spread in the CNS and to peripheral organs.

The prominence of the microgliosis and infiltrating inflammatory monocytes in the CNS of ZIKV-infected WT mice is also seen in mice infected intranasally with WNV [46, 59]. In WNV encephalitis, the disease is dependent on nitric oxide-producing monocytes/macrophages infiltrating the CNS parenchyma, while, similar to our results with the *Rag1*^*−/−*^ mice, CD4^+^ and CD8^+^ T cells do not contribute significantly to pathology in the absence of peripheral infection [59]. Of note, the number of macrophages infiltrating the CNS of ZIKV-infected WT mice at day 3 post infection was similar to the study by Getts et al. [59], suggesting that this monocyte/macrophage response is typical and likely to be dependent on IFN-Is [64]. The importance of monocytes and microglia in flavivirus encephalitis is also evident from a recent study in DENV encephalitis, where reduction of microglia had disease-enhancing effects [65]. This was accompanied by altered proliferation and activation of T cells suggesting immunoregulatory roles for microglia in DENV infection of the CNS. Together, these studies suggest a complex role for microglia and macrophages in flavivirus encephalitis that, dependent on the setting, may be disease-promoting or protective. Mechanistic details, however, remain to be clarified.

Previous studies have shown that in contrast to WT mice, mice deficient in the IFN-I system are highly susceptible to peripheral ZIKV infection [29, 31, 33, 53, 54]. Our findings demonstrate that in contrast to the periphery, the presence of a normal IFN-I system is insufficient to prevent virus replication and disease in the CNS following i.c. infection. Yet, the more rapid development of disease in *Ifnar1*^*−/−*^ mice compared with WT mice suggests some protective effects of IFN-Is in i.c.-infected WT mice. Thus, in contrast to WT mice, ZIKV rapidly spread within the brain and to the periphery in *Ifnar1*^*−/−*^ mice. This is further supported by high IFN-I mRNA levels in the CNS of WT and *Rag1*^*−/−*^ mice indicating that they contribute to virus control. Virus spread in *Ifnar1*^*−/−*^ mice was accompanied by significantly higher viral RNA levels at day 4 post infection, and it is conceivable that the cytopathic effects of ZIKV contributed to the faster onset of disease in *Ifnar1*^*−/−*^ mice compared with WT mice. Uncontrolled virus spread is also seen following peripheral infection of mice with deficiencies in the IFN-I system [31, 33] and in mice expressing human STAT2, which is recognized by ZIKV and degraded [35]. In these studies, mice not only show productive CNS infection but also develop a lethal encephalitis. We found that the encephalitis in ZIKV-infected *Ifnar1*^*−/−*^ mice was dominated by neutrophils, confirming a recent study in similarly infected IFN-I signaling deficient mice [58]. Neutrophils were mostly absent from the brains of WT mice at peak disease. Further, the infiltrating neutrophils were mostly immature as evident from the FlowSOM data, indicating a left-shift in the development of these cells in *Ifnar1*^*−/−*^ mice. Interestingly, both ZIKV encephalitis and influenza in *Ifnar1*^*−/−*^ mice following peripheral and pulmonary infection, respectively, are also characterized by the presence of neutrophils in the brain [64, 66]. Wang et al. further showed that in the absence of the IFN-I receptor, peripheral ZIKV infection resulted in a strong IL-1β production [66], which can be produced by neutrophils and is important to stimulate their activity (reviewed in [67]). IL-1β mRNA levels were also highly upregulated in the CNS, liver, and testis of our i.c.-infected *Ifnar1*^*−/−*^ mice indicating a similar mechanism. In contrast to neutrophils, inflammatory macrophages that dominated the CNS infiltrates in WT mice were only slightly increased in *Ifnar1*^*−/−*^ mice suggesting that IFN-Is are required to recruit these cells to the CNS following ZIKV infection. It is tempting to speculate that ZIKV induces a similar pathology in humans, that like *Ifnar1*^*−/−*^ mice lack a functional IFN-I system due to virus-mediated STAT2 degradation. However, it remains to be determined if CNS inflammation in ZIKV-infected humans is also dominated by neutrophils.

## Conclusions

In summary, we provide the first detailed characterization of an immune competent mouse model of ZIKV neuropathogenesis. Importantly, our findings demonstrate that in contrast to the periphery, the presence of a normal IFN-I system in the CNS is not sufficient to control ZIKV replication and subsequent disease. However, a defective IFN-I response enhanced virus replication, accelerated lethality, and shifted the inflammatory response from a monocyte/macrophage-dominated encephalitis that is independent of adaptive immune cells in WT mice to a neutrophil-dominated encephalitis in *Ifnar1*^*−/−*^ mice. Furthermore, our results also suggest a similar pathogenesis in ZIKV encephalitis to that seen in other neurotropic *Flaviviridae* including WNV and DENV.

## Additional files

Additional file 1: Gating strategy of flow cytometry analysis. (A) Gating strategy used for manual analysis applied to the transformed data used for computational analysis. (B) Overlays of manually gated populations on top of the tSNE plot. (TIF 4122 kb)

Additional file 2: Dual-label in situ hybridisation/histochemistry (ISH/HC). Photomicrographs of ISH against ZIKV combined with histochemistry against NeuN to detect neurons, GFAP to detect astrocytes or tomato lectin to detect microglia, monocytes/macrophages and blood vessels. Sections are from i.c.-infected mice at peak disease. Blue arrowheads indicate ISH-IHC-double positive cells, black arrowheads ISH-positive, IHC-negative cells and red arrowheads ISH-negative, IHC-positive cells. (TIF 13604 kb)

Additional file 3: tSNE plot of entire dataset coloured by FlowSOM cluster identities. (A) Every point in the tSNE plot represents a single cell. The centroid of each cluster is labelled with the corresponding cluster number. (B) Record of clusters that were combined or split to arrive at final populations. (C) tSNE plots of the same dataset coloured by the expression of all markers analysed. (TIF 4812 kb)

Additional file 4: Cell frequencies based on computational analysis of data and heatmap showing changes in cell number in each tSNE cluster. (A) Plots of the number of cells from specific populations per brain. Populations were identified using the clustering approach, and the cluster identities are listed in Additional file 2. Statistical comparisons of two groups were performed using a Mann-Whitney-Wilcoxon test for non-Gaussian data (also referred to as a ‘Wilcox test’ in R) using R. Overall variance of the dataset was assessed using a Kruskal-Wallis test for non-Gaussian data. (B) Heatmap showing the fold-change of the number of cells per cluster in each sample, relative to the average of WT mock-infected samples, were generated using a custom R script. Fold-change was plotted in log2, and coloured black (0 in log2, no change), red/yellow (an increase in fold change in log 2, >0 to greater than or equal to maximum value indicated on the scale bar), or blue (a decrease in fold change in log 2, <0 to less than or equal to minimum value indicated on the scale bar). Blue line graph overlaying the coloured scale bar indicates the relative proportion of data points that have a specific fold- change value. Columns are clustered together based on similarity, indicated by the coloured bars that group columns, determined by Euclidean distance. Rows were ordered manually. C) Overlays of clusters 1 (red), 2 (green), 12 (blue), and 17 (purple); representing different phenotypes of neutrophils. Top row shows data from ZIKV-infected WT mice, and the bottom row shows data from ZIKV-infected *Ifnar1*^*-/-*^ mice. (TIF 2056 kb)

## Data Availability

All relevant data generated or analyzed during this study are included in this published article.
