# Supplementary figures and images for: Zika virus encephalitis in immunocompetent mice is dominated by innate immune cells and does not require T or B cells
Source: J Neuroinflammation. 2019 Sep 11;16:177. doi: 10.1186/s12974-019-1566-5 (PMC6740023; doi:10.1186/s12974-019-1566-5)

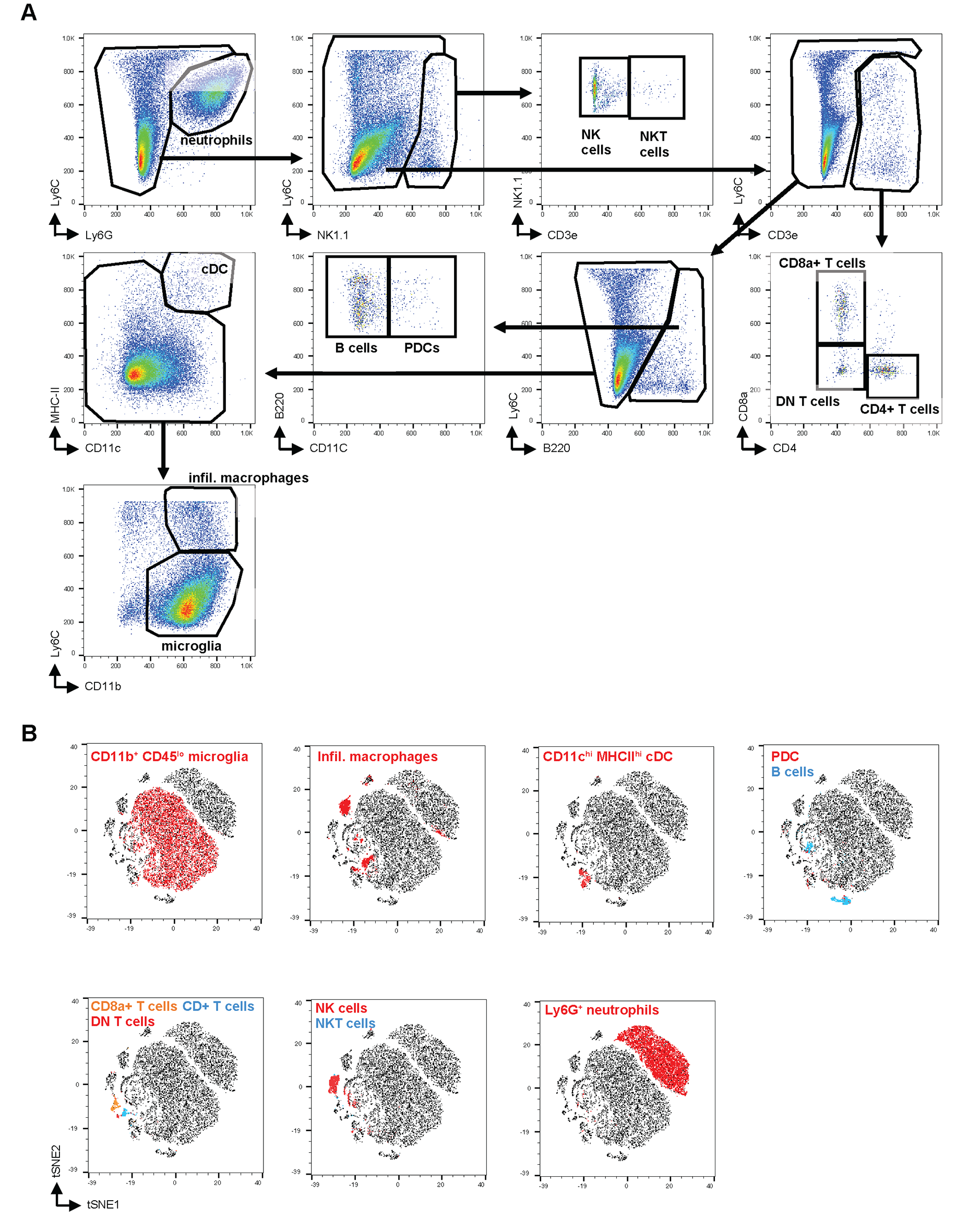

Supplement: Supplementary file 1 — Gating strategy of flow cytometry analysis. (A) Gating strategy used for manual analysis applied to the transformed data used for computational analysis. (B) Overlays of manually gated populations on top of the tSNE plot. (TIF 4122 kb) [file 12974_2019_1566_MOESM1_ESM.tif]

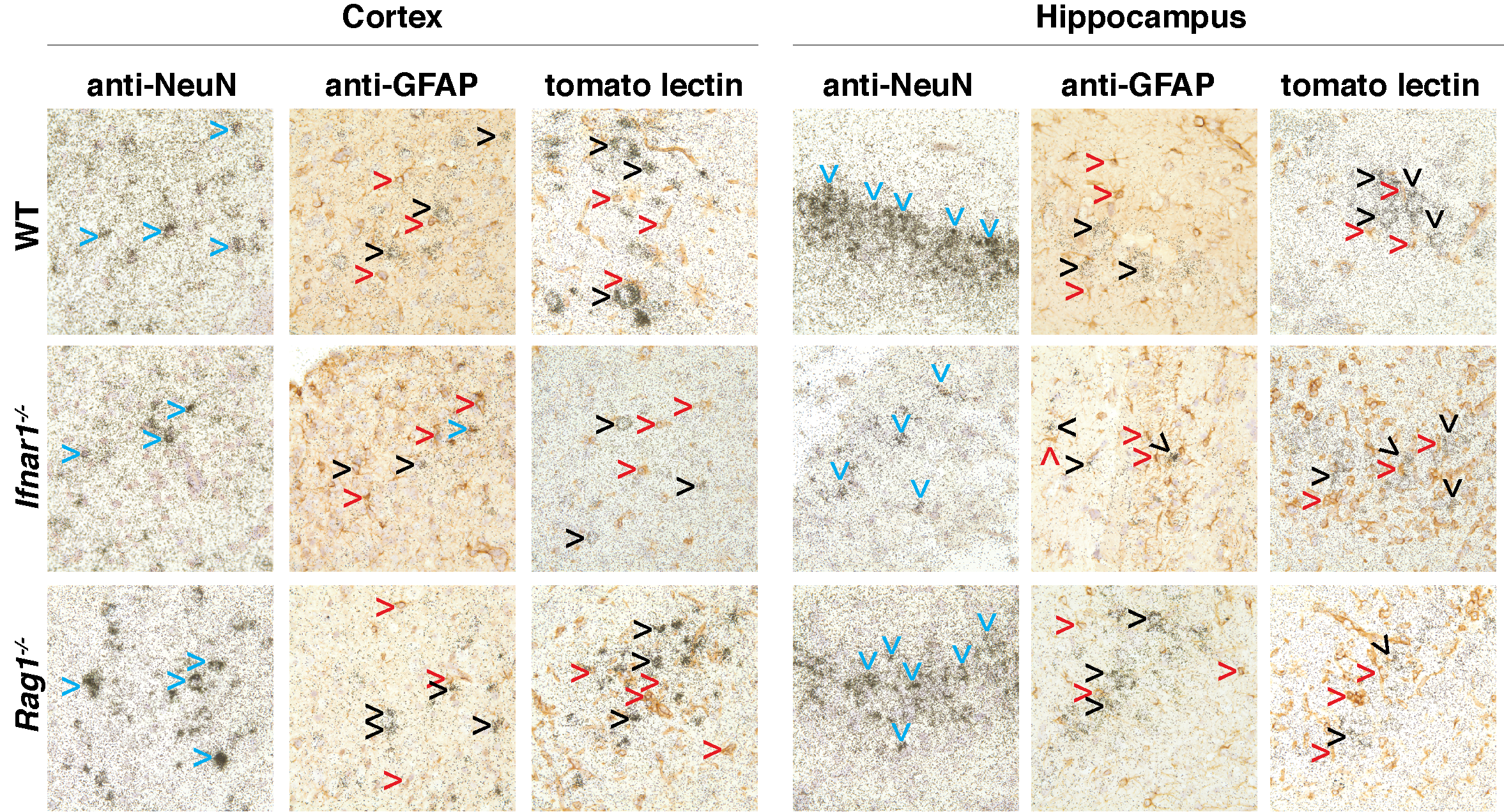

Supplement: Supplementary file 2 — Dual-label in situ hybridisation/histochemistry (ISH/HC). Photomicrographs of ISH against ZIKV combined with histochemistry against NeuN to detect neurons, GFAP to detect astrocytes or tomato lectin to detect microglia, monocytes/macrophages and blood vessels. Sections are from i.c.-infected mice at peak disease. Blue arrowheads indicate ISH-IHC-double positive cells, black arrowheads ISH-positive, IHC-negative cells and red arrowheads ISH-negative, IHC-positive cells. (TIF 13604 kb) [file 12974_2019_1566_MOESM2_ESM.tif]

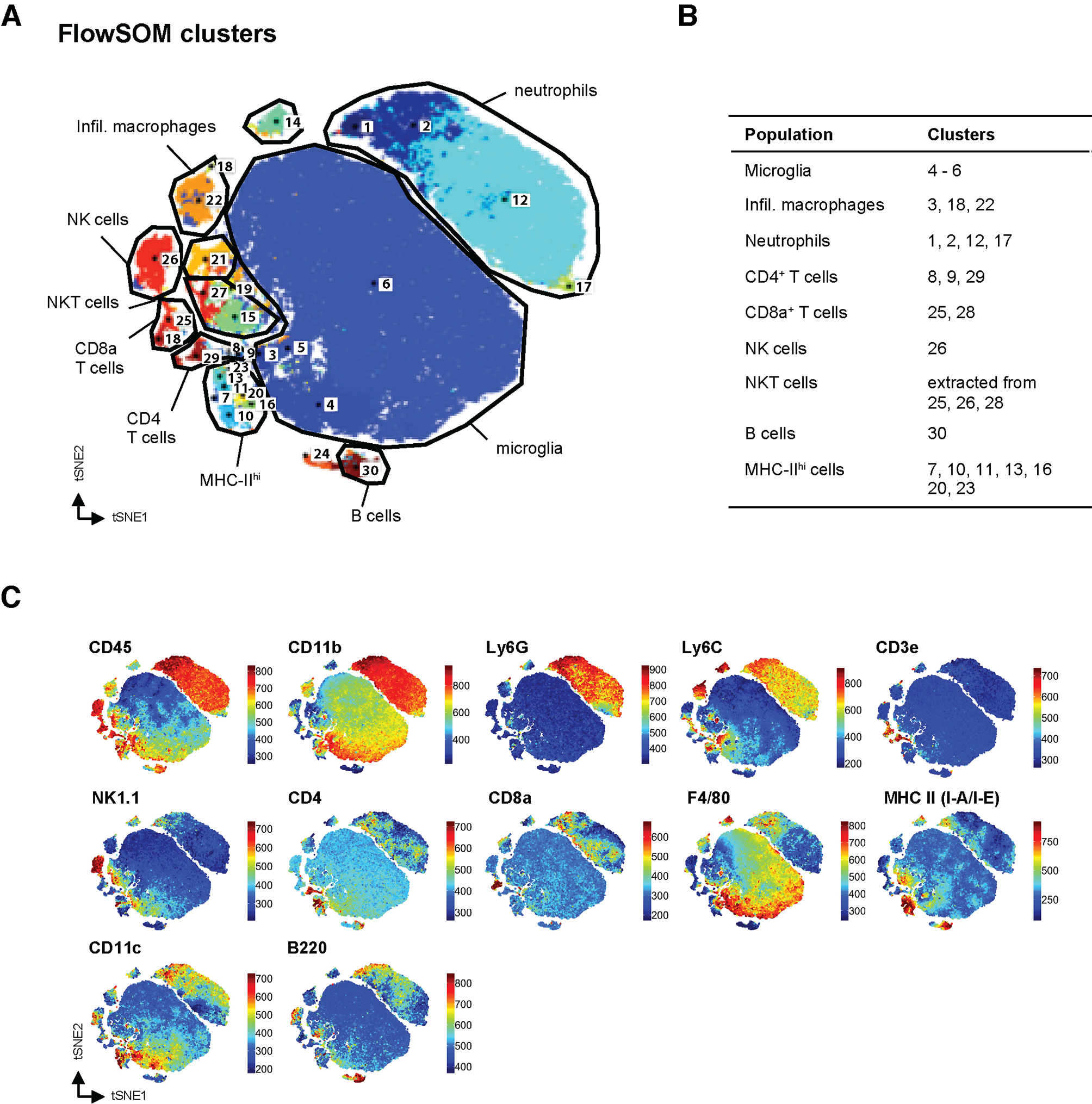

Supplement: Supplementary file 3 — tSNE plot of entire dataset coloured by FlowSOM cluster identities. (A) Every point in the tSNE plot represents a single cell. The centroid of each cluster is labelled with the corresponding cluster number. (B) Record of clusters that were combined or split to arrive at final populations. (C) tSNE plots of the same dataset coloured by the expression of all markers analysed. (TIF 4812 kb) [file 12974_2019_1566_MOESM3_ESM.tif]

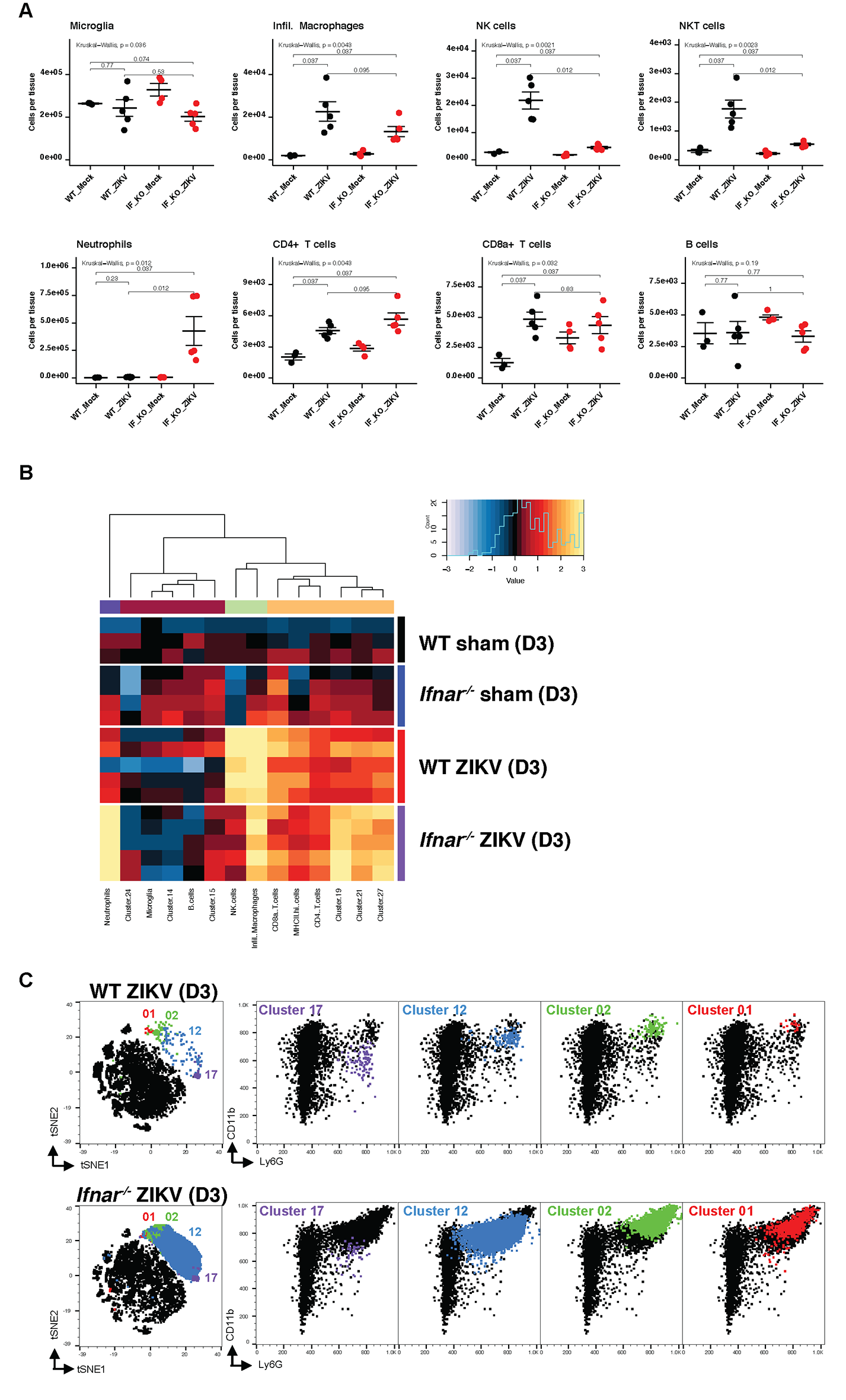

Supplement: Supplementary file 4 — Cell frequencies based on computational analysis of data and heatmap showing changes in cell number in each tSNE cluster. (A) Plots of the number of cells from specific populations per brain. Populations were identified using the clustering approach, and the cluster identities are listed in Additional file 2. Statistical comparisons of two groups were performed using a Mann-Whitney-Wilcoxon test for non-Gaussian data (also referred to as a ‘Wilcox test’ in R) using R. Overall variance of the dataset was assessed using a Kruskal-Wallis test for non-Gaussian data. (B) Heatmap showing the fold-change of the number of cells per cluster in each sample, relative to the average of WT mock-infected samples, were generated using a custom R script. Fold-change was plotted in log2, and coloured black (0 in log2, no change), red/yellow (an increase in fold change in log 2, >0 to greater than or equal to maximum value indicated on the scale bar), or blue (a decrease in fold change in log 2, <0 to less than or equal to minimum value indicated on the scale bar). Blue line graph overlaying the coloured scale bar indicates the relative proportion of data points that have a specific fold- change value. Columns are clustered together based on similarity, indicated by the coloured bars that group columns, determined by Euclidean distance. Rows were ordered manually. C) Overlays of clusters 1 (red), 2 (green), 12 (blue), and 17 (purple); representing different phenotypes of neutrophils. Top row shows data from ZIKV-infected WT mice, and the bottom row shows data from ZIKV-infected Ifnar1-/- mice. (TIF 2056 kb) [file 12974_2019_1566_MOESM4_ESM.tif]
